# Extracellular matrix remodeling in equine sarcoid: an immunohistochemical and molecular study

**DOI:** 10.1186/s12917-016-0648-1

**Published:** 2016-02-02

**Authors:** Manuela Martano, Annunziata Corteggio, Brunella Restucci, Maria Ester De Biase, Giuseppe Borzacchiello, Paola Maiolino

**Affiliations:** Department of Veterinary Medicine and Animal Productions, Naples University “Federico II”, Via F. Delpino 1, 80137 Naples, Italy; Present Address: Institute of Protein Biochemistry (IBP) National Research Council (CNR), Via Pietro Castellino 111, 80131 Naples, Italy

**Keywords:** BPV, ECM, Equine sarcoid, MMPs

## Abstract

**Background:**

Equine sarcoids are locally invasive, fibroblastic benign skin tumors. Bovine papillomavirus type-1 (BPV-1) and/or Bovine papillomavirus type-2 (BPV-2) are believed to be the causative agent of sarcoids, although the mechanisms by which the virus induce the tumor are still poorly understood. We hypothesized that in genetically predisposed equines latent BPV infection may be reactivated by immunosoppression and/or mechanical injury leading to a form of pathologic wound which may transform into a sarcoid. In this study, we investigated in 25 equine sarcoids and in five normal skin samples the histological features and evaluated the immunohistochemical and molecular expression of type I and type III Collagen, vimentin (VIM), alfa Smooth Muscle Actin (α-SMA), Matrix Metalloproteinase (MMPs) -2, 9, 14 and tissue inhibitor of metalloproteinase 2 (TIMP-2).

**Results:**

In 64 % of investigated sarcoids, type I collagen staining was stronger than that of type III collagen. In 80 % of sarcoids, SFs were strongly positive for vimentin and negative for α-SMA; the remaining sarcoid samples (20 %) showed 70–80 % of SFs labeled for vim and approximately 20–30 % labeled for α-SMA. Moreover, all sarcoid specimen showed a variable staining pattern (weak to moderate) for MMP-9 and MMP-14, and a moderate to strong staining for MMP-2 and TIMP-2. Biochemical analysis confirmed immunohistochemical results and showed in sarcoids, for the first time, the cleaved form of MMP9, the 35 KDa active species for MMP-9.

**Conclusions:**

This study revealed that in equine sarcoids exhibit an altered turnover of the Extracellular Matrix (ECM) deposition and degradation, as result of an altered expression of MMPs and TIMPs. Therefore, these observations seem to confirm that the basic mechanism for growth of equine sarcoids could be a neoplastic transformation during wound healing.

## Background

Equine sarcoids are locally invasive, fibroblastic benign skin tumors and represent the most common skin tumor in equidae worldwide [1, 2]. They can occur as single lesion, or, more commonly, as multiple lesions, frequently at sites of previous injury and scarring; although they can develop anywhere on the integument sites of predilection are in particular the paragenital region, the thorax–abdomen and the head [3, 4]. Equine sarcoids rarely regress, are notoriously difficult to treat and are associated with a high recurrence rate following surgical intervention [5–7]; these features are likely due to the invasiveness of sarcoid fibroblasts (SFs) [8, 9]. BPV-1 and less commonly BPV-2 are widely recognized as the causative agents of the disease. Although the viral etiology, the biology, the morphology and the epidemiology of equine sarcoids are known [4, 10, 11], the pathogenic events leading to the development of tumour and the mechanisms used by BPV to induce the tumour are less understood. Sarcoid formation is known as one of the main long-term complications in the wound healing of horses [12, 13]. We hypothesized that in healthy genetically predisposed horses, BPV-1/BPV2 may be responsible for abnormal fibroblast proliferation on one hand, and on the other for alterations in dynamics of the extracellular matrix (ECM) and its main components (e.g. collagen). These changes could induce an alteration of the wound healing process and may therefore be an important factor in the pathogenesis of equine sarcoids. The hypothesis that cancer may be “a wound that won’t heal” has been supported by numerous studies [14–16] suggesting that wound healing and tumorigenesis share consistently similarities in terms of histological features and signaling molecules; those are among others Matrix Metalloproteinases (MMPs), a family of at least 25 zinc-dependent endopeptidases, and their inhibitors (TIMPs) all essentially capable of degrading Extracellular Matrix (ECM), including collagen. Besides participating in normal connective tissue homeostasis and remodeling, MMPs activity is involved in remodeling of ECM and the migration of numerous cell types during various pathological conditions, such as wound healing, keloid formation, chronic inflammatory diseases, and as well as in tumour invasion [17–19]. In this study, we speculate that changes of the expression levels and of the enzymatic activity of MMP-2, MMP-9, MMP-14 (MMP1-MMT) and TIMP-2 may play an important role in the pathogenesis of sarcoids, being responsible for ECM turnover. Of the growing family of MMPs, MMP-2 (gelatinase A, 72-kDa type IV collagenase,) and MMP-9 (gelatinase B, 92-kDa type IV collagenase) are unique for their fibronectin-like collagen binding domains and are responsible of degradation of type IV collagen in the basement membranes and in fibrillar collagens, which are essential features of tissue repair and remodeling processes. Their activity is controlled by a group of protein inhibitors, the TIMPs. MMP-14 is a trans-membrane protease, capable of degrading different ECM components such as collagen types I, II, and III, as well as fibronectin and laminin [20]. The main interest in this enzyme is due to its ability to activate different proteases, particularly MMP-2 and MMP-9 [21]. Recently, Yuan et al. (2010) [8] have shown that BPV-1 induce overexpression of MMPs contributing to invasiveness of SFs in vitro. Our in vivo study aimed at gaining new insights into the pathogenetic mechanisms of equine sarcoids, by employing immunohistochemistry and western blot analysis to investigate their histological features, as well as the expression of type I and type III collagen, MMP-2, MMMP-9, MMP-14 and their inhibitors, such as TIMP-2. Moreover, the enzymatic activities of MMP-2 and MMP-9 were quantified by gelatin-zymography of the same homogenized tumour tissues.

## Results

### Histological features

**Fig. 1:**
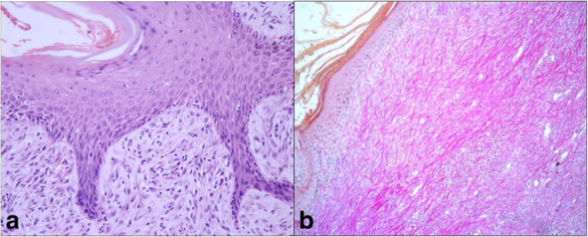
**a** Equine sarcoid. Epidermal hyperplasia, rete peg and picket fence formation. Hematoxylin-eosin. 20X. **b** Equine sarcoid. Red color collagen fibers (collagen I) in equine sarcoid. Van Gieson Stain. 10X

Examined sarcoids showed the typical histological changes in their epidermal (when present) and dermal component such as hyperkeratosis and epidermal hyperplasia often accompanied by rete pegs extending deep into the proliferating dermal connective tissue. Dermal proliferation consisted of tightly whirling plump spindle cells, proliferating in an ECM which appeared more developed than normal. The superficial dermal fibroblasts were usually oriented perpendicular to the basilar epidermal layer in a ‘picket fence’ pattern (Fig.1a). Ulceration as well as inflammation (infiltration of polymorphonuclear cells) were commonly seen. Van Gieson’s stain of sarcoids confirmed an increase in the amount of deep red color collagen fibers in the dermis, identified as mature collagen (Type I), compared with pink color collagen fibers, identified as immature collagen (Type III) (Fig.1b), as compared to normal skin.

### Immunohistochemistry

The expression patterns of Vimentin (VIM), alpha-Smooth Muscle Actin (α-SMA), type I Collagen, type III Collagen, MMP-2, MMP-9, MMP-14, and TIMP-2 in 25 equine sarcoids and five normal skin samples are summarized in Table 1.

**Table 1:** Immunoreactivity scoring of VIM, α-sma, Type I collagen, Type III collagen, MMP-2, MMP-9, MMP14, TIMP-2 in 25 equine sarcoids and 5 normal skin

Samples^a^	Location	VIM^b^	Α-SMA^b^	Type I collagen^b^	Type III collagen^b^	MMP-2^b^	MMP-9^b^	MMP14^b^	TIMP-2^b^
T1	Abdomen	++	-	+	+	+	+	+/−	++
T2	Limbs	++	-	+	+	++	+	+/−	++
T3	Neck	++	-	+	+	+	+	+/−	++
T4	Abdomen	++	-	+	+	+	+/−	+/−	++
T5	Paragenit region	++	-	+	+	++	+	+	++
T6	Pectoral region	++	-	+	+	+	+/−	+	++
T7	Paragenit region	++	-	+	+	+	+/−	+	++
T8	Neck	++	-	+	+	++	+	+	++
T9	Neck	++	-	+	+	+	+/−	+/−	++
T10	Pectoral region	++	-	++	+	+	+	+/−	++
T11	Paragenit region	++	-	++	+	++	+	+/−	++
T12	Limbs	++	-	++	+	++	+	+/−	++
T13	Neck	++	-	++	+	+	+/−	+	++
T14	Abdomen	++	-	++	+	+	+/−	+	++
T15	Limbs	++	-	++	+	++	+	+/−	++
T16	Pectoral region	++	-	++	+	+	+/−	+/−	++
T17	Pectoral region	++	-	++	+	+	+/−	+/−	++
T18	Limbs	++	-	++	+	++	+	+	++
T19	Abdomen	++	-	++	+	+	+/−	+	+
T20	Pectoral region	++	-	++	+	+	+/−	+/−	+
T21	Limbs	++	++	++	+	++	+	+/−	+
T22	Neck	++	++	++	+	++	+	+/−	+
T23	Abdomen	++	++	++	+	+	+/−	+	+
T24	Paragenit region	++	++	++	+	+	+/−	+	+
T25	Pectoral region	++	++	++	+	+	+/−	+	+
N1	Limbs	n.a.	n.a.	+/−	+/−	+	+/−	-	-
N2	Abdomen	n.a.	n.a.	+/−	+/−	+	+/−	+/−	+/−
N3	Pectoral region	n.a.	n.a.	+/−	+/−	+	+/−	-	+/−
N4	Neck	n.a.	n.a.	+/−	+/−	+	+/−	+/−	-
N5	Limbs	n.a.	n.a.	+/−	+/−	+	+/−	+/−	+/−

^a^ *T* tumour sample, *N* normal skin sample, ^b^- negative staining; +/− weak immunolabelling; + moderate immunolabelling; ++ extensive and strong immunolabelling; *n.a.* not assessed

#### Normal skin

All normal skin samples showed positive immunostaing for type I and III Collagen, which was light brown stained with a widely distributed staining pattern within the dermal layer. Moreover, a weak and finely granular cytoplasmic MMP-2 and MMP-9 reactivity was observed in the epidermis. TIMP-2 and MMP-14 immunoexpression was present in the epidermis but also in vascular endothelial cells, inflammatory cells and fibroblastic cells.

#### Sarcoid samples

**Fig. 2:**
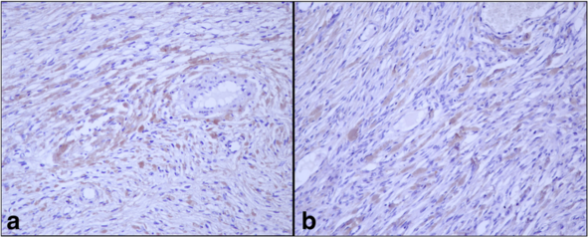
**a** Type I Collagen and **b** Type III collagen immunostaining in equine sarcoids. Streptavidin-biotin-peroxidase stain 20X

**Fig. 3:**
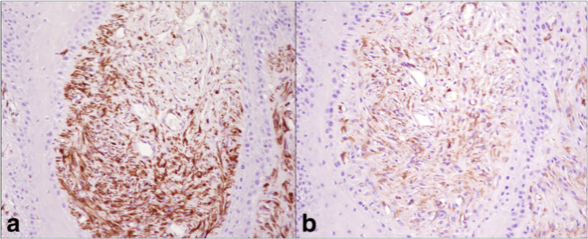
VIM and Alfa-sma immunostaining in equine sarcoid. **a** 70–80 % of SFs show strong vimentin immunostaining; **b** 20–30 % of SFs show strong Alfa-sma immunostaining. Streptavidin-biotin-peroxidase stain 20X

**Fig. 4:**
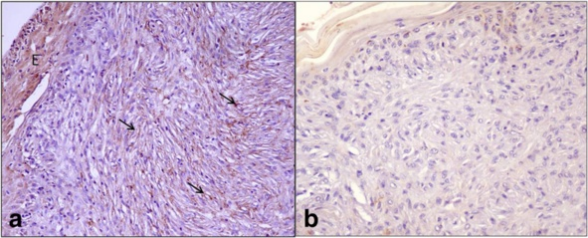
**a** MMP-2 immunostaining in equine sarcoid. SFs (*arrows*) show a strong immunostaining, the epidermis (E) is also strongly MMP-2 positive. **b** Secondary-only negative control. Streptavidin-biotin-peroxidase stain. 20X

**Fig. 5:**
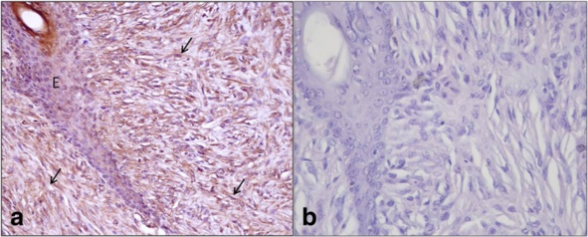
**a** MMP-9 immunostaining in equine sarcoid. The epidermis (E) shows strong positivity except for rete peg epithelium, SFs are strongly MMP-9 positive (*arrows*). **b** Secondary-only negative control. Streptavidin-biotin-peroxidase stain. 20X

**Fig. 6:**
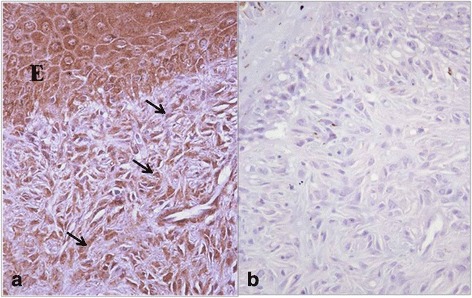
**a** TIMP-2 immunostaining in equine sarcoid. TIMP-2 is strongly expressed by epidermis (E) and by 50–70 % of SFs (*arrows*). **b** Secondary-only negative control. Streptavidin-biotin-peroxidase stain. 40X

**Fig. 7:**
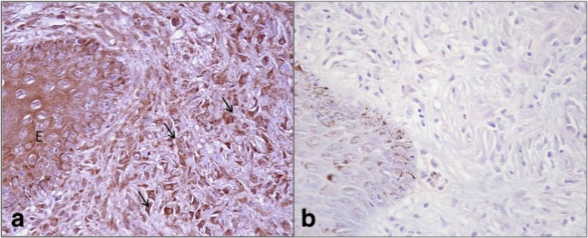
**a** MMP-14 immunostaining in equine sarcoid. MMP-14 is strongly stained by epidermis (E) and by 50–70 % of SFs (*arrows*). **b** Secondary-only negative control. Streptavidin-biotin-peroxidase stain. 40X

Type I and Type III collagen appeared as fine discontinuous individual fibers in a loose network, and showed a moderate immunosignal in 36 % of sarcoid samples (Fig. 2: a-b). Type I Collagen staining was stronger than type III collagen in the remaining samples (64 %). In 80 % of sarcoids, SFs were strongly positive for vimentin and negative for α-SMA; in the remaining samples (20 %), 70–80 % of SFs were labeled for vimentin (Fig. 3a) and approximately 20–30 % were strongly labeled for α-SMA (Fig. 3b). Furthermore, sarcoid specimens showed a variable (64 % moderate; 36 % strong) and finely granular staining pattern for MMP-2 in 30–50 % of SFs, as well as in the cytoplasm of epidermal cells (Fig. 4a). Sarcoids featured a weak (52 %) to moderate (48 %) staining for MMP-9, which appeared highly and finely granular in the cytoplasm of epidermal cells and rarely (often lacking) in the rete peg epithelium. A moderate to strong cytoplasmic staining for MMP-9 was observed in 30–70 % of SFs, inflammatory cells and vascular endothelial cells (Fig. 5a). TIMP-2 showed extensive and strong cytoplasmic positivity in epidermal cells and in 50–70 % of SFs in almost every sample (72 %) (Fig. 6a). In all tumors, MMP-14 expression was observed in epidermis and in 50–70 % of SFs, with variable (56 % weak; 44 % moderate) cytoplasmic immunolabelling (Fig. 7a).

### Biochemical analysis

**Fig. 8:**
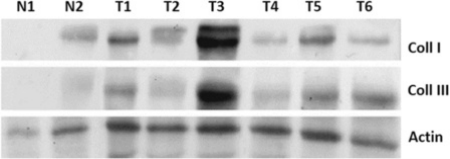
Collagen type I and type III protein expression in equine sarcoids (T) and normal skin (N). Collagen type III are expressed in higher amount in sarcoids (T) when compared to normal skin samples (N). Actin protein levels confirm the amount of protein loading in each lane

**Fig. 9:**
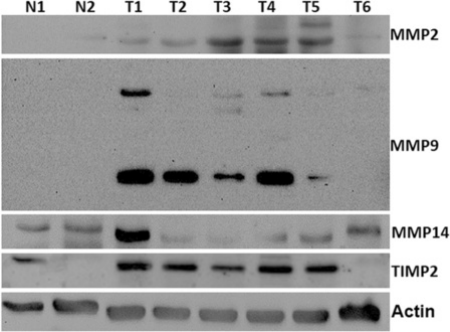
MMP-2, MMP-9, MMP-14 and TIMP-2 protein expression in equine sarcoids (T) and normal skin (N). MMP-2 and MMP-9 are expressed in higher amount in sarcoids. MMP9 is present in two different forms, a 82 kDa band and 35 kDa band (super active form). MMP-9 is expressed at similar levels in all the analyzed samples, albeit in T1 samples is present at very higher level. TIMP-2 is expressed in higher amount in sarcoids (T) when compared to normal skin samples (N). Actin protein levels confirm the equal amount of protein loading in each lane

**Fig. 10:**
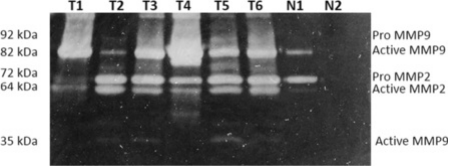
MMP-2 and MMP-9 protein expression and activation. Gelatin zymography was performed from equine sarcoids (T) and normal skin (N) tissues homogenates supernatants. In all sarcoids the active form of MMP-9 (82 kDa) is the predominant form, and a super active form (35 kDa) is present in the same samples. MMP-2 is present in both proform (72 kDa) and active form (62 kDa) in all sarcoids

To further confirm our findings, six sarcoid samples, which were available for biochemical analysis and two skin samples from healthy horses were subjected to western blot analysis. Hela cell line was also analyzed as positive control for the antibodies used (data not shown). The anti-Collagen I and anti-Collagen III antibodies yielded a band of the expected molecular weight in the neoplastic tissues and normal skin. An increase in the amount of both collagen type protein levels in all tumour samples compared with normal skin was observed, albeit in different amounts among the samples (Fig. 8). The analysis of MMP-2 showed that 50 % of sarcoid samples (T3, T4 and T5) had a high level of protein, above all in its active (cleaved) form (62 kDa). In addition, an increase in the expression level of MMP9 in all tumour samples versus healthy skin samples was observed. The sarcoids showed the overexpression of the cleaved form of MMP9, the 35 KDa active species which was an autocatalytic product of the 82KDa pre-form. We analyzed the expression of MMP-14 and TIMP-2, in order to evaluate their involvement in MMP2 activity. There was no significant up-regulation of MMP14 protein in the sarcoids compared to normal skin samples, even if in one sarcoid sample (T1) it was present at a very high level. TIMP2 was overexpressed in sarcoids when compared to normal skin. Actin was shown as control for ensuring the equal loading of protein extracts (Fig. 9). Finally, we examined gelatinase MMP’s activity in sarcoid tumors using zymography, which has been extensively used to detect both latent and active form of MMPs. Gelatine zymography was employed to specifically detect MMP-2 and MMP-9 protease activity. As shown in Fig. 10, all the examined sarcoids expressed the pro- and active form of MMP2. However, sarcoids showed higher expression levels than normal skin samples. In addition, the MMP9 was present in the activated form in all the analyzed sarcoids. Interestingly, four out of six the sarcoid samples (80 %) overexpressed a cleaved form of 35 kDa MMP9, thus demonstrating the strong activity of MMP9 during sarcoids tumorigenesis.

## Discussion

Sarcoids are the most common equine skin tumours, characterized by neoplastic fibroblasts intermingled in a collagenous stroma, frequently associated to epidermal hyperplasia [2, 22]. BPV-1/BPV-2 are believed to be the causative agent of equine sarcoid. This is based on the fact that: 1) BPV-1/-2 DNA is detected in the majority of sarcoid tumors [7, 23–27]; 2) BPV genes are expressed in sarcoids [10, 28–30]; 3) experimental inoculation of equine skin with BPV induces sarcoid-like lesions in horses [31]; 4) BPV- 1 DNA can transform primary equine fibroblasts in vitro [8, 32]. Interestingly, BPV DNA presence has also been reported in normal skin and the virus has been found to be transcriptionally active in some cases of equine inflammatory skin lesions [33, 34]. It is noteworthy that very recently BPV has been found also in exuberant granulation tissue [35]. It is widely accepted that equine sarcoids may develop subsequently to injury and scarring in genetically predisposed equines [24]. For this reason we hypotized that latent BPV infection may be reactivated by chronic physical trauma, leading to development of a form of pathologic wound healing (e.g. keloid); thus, the scar producing process may be altered during the maturation phase of wound healing allowing transformation of scar tissue (keloids) into sarcoids.

In fact, our immunohistochemical results showed that, in most sarcoids (20/25), fibroblasts represented the principal cellular population, the remainder was composed of fibroblasts and myofibroblasts.

Furthermore, in our sample tissues collagen content was elevated and disorganized when compared to normal skin and, in contrast to what Williams et al. reported [36], the ratio of type I (mature collagen) to type III collagen (immature collagen) seemed to be slightly higher, which was as also demonstrated by Van Gieson stain. Also, and in line with the immunohistochemical results, western blotting analysis confirmed that collagen I and III were present in higher amounts in tumour samples when compared to normal skin samples. It is known tissue of normal wound repair contains primarily type III collagen with abundant myofibroblasts. In contrast, abnormal wound repair tissue, (for instance keloid), consists of type I and III bundles with few myofibroblasts [37]. Combining these data strongly suggests that these sarcoid tumours actually originate in abnormal wound repair tissue [38].

We hypothesized that the basic mechanism for the development of equine sarcoids could be an imbalance of ECM deposition and degradation as seen also during pathologic wound healing.

This process is mediated by ECM degrading enzymes, such as MMPs, and we believe that these changes are likely the result of altered expression levels between these enzymes and their inhibitors (TIMPs). The MMPs are usually not detectable or at very low levels in healthy resting tissue and are instead induced in wound repair and in keloid formation in response to cytokines, growth factors and/or cell contact with ECM [17–19]. It has been shown that the over-expression and activation of MMPs is induced by BPV oncoproteins in equine sarcoid fibroblasts and, recently, MMPs expression has been confirmed also in vivo [8, 9, 32]. These previous observations are in line with the results of our study in which we also found that both MMP-9 and MMP-2 were consistently expressed by epidermal and dermal cells and with different intensity.

MMP-9 is secreted as pro-MMP-9 (92 kDa) and is activated into the functional form (82 kDa).

In equine sarcoids our data show for the first time, the overexpression of a 35 KDa super-active form of MMP-9, an auto-catalytic product of the 82KDa proform, which possesses highly efficient proteolytic activity for different ECM proteins (gelatins, fibronectin and collagen type IV) [39]. In our sarcoid samples, MMP-9 was expressed by SFs and keratinocytes, strongly suggesting its role in the formation of long rete pegs [40, 41]; this in turn promotes keratinocyte detachment from the basement zone through controlled digestion of type IV collagen.

Thus, sarcoid development could be the result of fibroblast stimulated proliferation of overlying epithelial cells (with rete peg formation), which in turn stimulate the fibroblasts in the underlying dermis to proliferate and to produce more collagen.

In our study, MMP-2 showed a variable and finely granular staining pattern in 30–50 % of SFs, confirming that fibrillar collagens, rather that collagen IV, are its specific substrate.

Normally, MMP-2 is secreted as pro-MMP-2 (72 kDa) and is activated into the functional form (66 kDa). Biochemical analysis showed that MMP-2 was overexpressed and hyper-activated in 50 % of sarcoid samples, when compared to normal skin. Moreover, in sarcoids MMP-2 expression was associated with a weak to moderate expression of MMP-14, which is considered its main activator; this, with a strong expression of TIMP-2, which is considered its main inhibitor [42]. Although MMP-14 expression seems increased, the higher level of TIMP-2, could lead to a decrease in MMP2 collagenolytic activity, which in turn causes insufficient degradation of collagen produced in excess by SFs. In fact, in our sarcoid samples a high content of collagen(s) was observed, suggesting that ECM deposition continues with insufficient degradation.

Therefore, we postulate that excessive and progressive deposition of connective tissue (collagen) in sarcoids, as well as in keloids [43], might not only be the result of elevated synthesis by SFs, but also caused by a deficiency in matrix degradation due to an alterated expression of MMPs and TIMPs. This imbalance between production and degradation of collagen could play an important role in the pathogenesis of the equine sarcoid. Therefore, it may be suggested that, in genetically predisposed equines with latent BVP infection, an altered wound healing process creates a microenvironment that activates the latent infection, leading to neoplastic transformation and sarcoid formation.

## Conclusions

Currently, there is no efficient curative therapy for equine sarcoids. The commonly employed treatments include cryotherapy, surgical excision and local immune modulation [44]. The present findings include the identification of the main cellular effectors of sarcoid growth, that is, the key cytokines regulating the scar formation process, and the regulators of ECM turnover; these findings therefore open the avenue for a number of potential therapeutic approaches that are likely to be developed in the near future.

## Methods

### Tumour samples

Ethics approval was obtained from the Ethical Animal Care and Use Committee of the University of Naples Federico II (Prot. N° 125861; 23/12/2015).

Twenty-five samples of equine sarcoid (each from a different horse), were clinically identified based on their gross morphology according to Pascoe and Knottenbelt (1999) [45]. Tumors were localized on the abdomen (5), neck (5), (para)-genital (4), pectoral region (6), and limbs (5) (Table 1). The length of time the sarcoids were present was variable (from 1 month to over 6 years), and often not precisely known.

No history of previous skin lacerations (wounds) could be determined due to lack of informations from both owners and practioneers. Sarcoid tissues used in this study were known to be positive for BPV 1- BPV 2 DNA ([28]; personal observations). Tumors, together with five normal skin samples from healthy horses, were either surgically excised or a representative biopsy was taken under local anesthesia. In all cases, owners signed a written consent form following a detailed verbal explanation of the study protocol. Samples were 10 % formalin fixed, paraffin-embedded for routine histological processing and stained with haematoxylin and eosin for light microscopy study and Verhoeff-Van Gieson (VVG) method to asses collagen content.; six out of 25 sarcoid samples were perfused thoroughly with cold 0.9 % NaCl and frozen at −80 °C for western blotting analysis.

### Immunohistochemistry

Paraffin sections of 25 sarcoids and five normal skin from healthy horses were dewaxed in xylene, dehydrated in graded alcohols and washed in 0.01 M phosphate-buffered saline (PBS), pH 7.2–7.4. Endogenous peroxidase was blocked with hydrogen peroxide 0.3 % in absolute methanol for 30 min. The immunohistochemical procedure (streptavidin biotin- peroxidase method, (LSAB Kit; Dako, Glostrup, Denmark) was the same as that used by the authors in a previous study [46]. Primary antibodies, used in this study are listed in Table 2. Antibodies were diluted in an antibody diluent (Dako, Glostrup, Denmark) and applied overnight at 4 °C. The immunolabelling procedure included negative control sections incubated with PBS instead of the primary antibody. A mixture of biotinylated anti-mouse and anti-rabbit immunoglobulins (LSAB Kit; Dako), diluted in PBS, was used as secondary antibody, and applied for 30 min. After washing in PBS, the sections were incubated in streptavidin conjugated to horseradish peroxidase in Tris–HCl buffer containing sodium azide (LSAB Kit; Dako) 0.015 %, for 30 min. To reveal immunolabelling, diaminobenzidine tetrahydrochloride was used as a chromogen, and haematoxylin was used as counterstain.

**Table 2:** List of primary antibodies used for immunohistochemistry and western blotting analysis

*Antibody*	*Manufacturer*	*Clone*	*Specificity*	*Host species*	*Antigen retrieval*	*IHC Dilution*	*WB Dilution*
VIMENTIN	Dako cytomation	V-9	man, cow, dog, hamster, horse, rabbit, rat	mouse	No antigen retrieval	1:50	-
SMOOTH MUSCLE ACTIN	Dako cytomation	1-a-4	chicken, cow, rat	mouse	Citrate, ph 6.0, 30 min, steamer	1:50	1:5000
TYPE I COLLAGEN	Ab-cam	Col-1	rat, rabbit, cow, human, pig, deer	mouse	Citrate, ph 6.0, 30 min, steamer	1:100	1:5000
TYPE III COLLAGEN	Millipore	Ie7-d7	rabbit, human	mouse	Citrate, ph 6.0, 30 min, steamer	1:100	1:5000
MMP-2	Thermo-scientific	*Ab-7*	human, mouse, rat, cow	rabbit	Citrate, ph 6.0, 30 min, steamer	1:200	1:500
MMP-9	Millipore	56-2a4	human, rat, rabbit, guinea pig	mouse	Citrate, ph 6.0, 30 min, steamer	1:200	1:1000
MT1-MMP (MMP-14)	Millipore	113-5b7	human	mouse	Citrate, ph 6.0, 30 min, steamer	1:200	1:500
TIMP-2	Millipore	2TMP05	bovine, guinea pig, human, mouse, rat, rabbit	mouse	Citrate, ph 6.0, 30 min, steamer	1:200	1:200

### Scoring of immunoreactivity

The intensity of immunolabelling in each specimen, for each antibody, was scored by two independent observers (PM, MM) under blinded conditions, as performed in a previous study [47]. For each tumor 20 fields were examined at 200X magnification (20X objective 10X ocular), and immunosignal was scored from absent to strong, as follows: n.a., not assessed; − negative staining; +/− weak immunolabelling; + moderate immunolabelling; ++ extensive and strong immunolabelling.

### Protein extraction and SDS PAGE/Western blotting

Six sarcoids (T1, T2, T3, T4, T5, T6), two samples of normal skin (N1, N2) and Hela cells line (positive control) were available for molecular analysis. Tissues were snap frozen in liquid nitrogen and homogenized in ice-cold lysis buffer (50 mM tris PH 7.5, 150 mM NaCl, 1 % Triton, 0.25 % Deoxicolic acid, 1 mM EDTA) added with protease inhibitor cocktail (Sigma, Milan, Italy). HeLa cell lines were grown for 2 days in 60-mm dishes, washed with ice-cold phosphate saline buffer two times and lysed for 20 min in ice-cold lysis buffer. Tissue homogenates and cell lysates were clarified by centrifugation and protein concentration was determined by Bradford protein assay performed according to manufacturer protocol (Bio-Rad Laboratories, Milan, Italy). 50 μg of total protein were boiled at 100° for 5′ in Laemmli sample buffer (Bio-Rad Laboratories, Hercules, CA) and analyzed by SDS polyacrylamide gel electrophoresis (PAGE). The proteins were blotted from the gel onto nitrocellulose membranes. The membranes were blocked for 1 h with 5 % bovine serum albumin (BSA) at room temperature and incubated with anti-MMP2 (1:500), anti-MMP9 (1:1000), anti-MMP14 (1:500), anti-TIMP-2 (1.200), anti-collagen I (1:5000), and anti-collagen III (1:5000) antibodies (Table 2). After appropriate washing steps, peroxidase-conjugated anti-rabbit or anti-mouse IgG (1:2000, Santa Cruz Biotechnology), were applied for 1 h at room temperature. After washing, bound antibody was visualized on enhanced chemiluminescence (ECL) film (Amersham Pharmacia Biotech). The blots were stripped and reprobed against mouse anti-actin antibody (Santa Cruz Biotechnology) at 1:5000 to confirm equal loading of proteins in each lane. For collagen I and III protein detection the SDS-PAGE procedure was performed in non-denaturing conditions.

### Gelatinase zymography

Substrate-specific zymography for determination of gelatinolytic activity of MMP-2 and MMP-9 was performed as previously described [48]. Briefly, 20 μg of each protein extract were subjected to gel electrophoresis using 10 % Zymogram (Gelatin) pre cast Gel and zymogram gel was developed according to manufacturer protocol (Bio-Rad Laboratories, Milan, Italy). After electrophoresis, the gel was washed twice 2.3 % triton X-100 for 30 min and incubated in development buffer (50 mM tris, 200 mM NaCl, 5 mM CaCl_2_, 0.02 % Brij-35 at 37 °C for 20 h. Gel was then stained with 0.5 % coomassie blue R-250 in staining solution (40 % methanol, 10 % acetic acid, 0.5 % coomassie blue, 100 ml deionized water) at room temperature (RT) for 1 h and was de-stained in destaining solution (40 % methanol, 10 % acetic acid, 250 ml deionized water), until clear lysis bands appeared. To quantify the intensities of the degradated bands, zymogram gel was scanned using ChemiDoc gel scanner (Bio-Rad Laboratories).

### Availability of supporting data

The data sets supporting the results of this study are included in the article and its additional files.

## Abbreviations

ECM: extracellular matrix
MMP: matrix metalloproteinase
TIMP: tissue inhibitor of metalloproteinase
SFs: sarcoid fibroblasts
BPV-1: bovine papillomavirus type-1
BPV-2: bovine papillomavirus type-2
VIM: vimentin
α-SMA: alpha-smooth muscle actin
T: sarcoid samples
N: normal skin
